# Backbone resonance assignments of the catalytic and regulatory domains of Ca^2+^/calmodulin-dependent protein kinase 1D

**DOI:** 10.1007/s12104-020-09950-x

**Published:** 2020-06-13

**Authors:** Michael H. G. Tong, Mark Jeeves, Sundaresan Rajesh, Christian Ludwig, Marc Lenoir, Jitendra Kumar, Darren M. McClelland, Fedor Berditchevski, Julia A. Hubbard, Colin Kenyon, Sam Butterworth, Stefan Knapp, Michael Overduin

**Affiliations:** 1 Institute of Cancer and Genomic Sciences, University of Birmingham, Edgbaston, Birmingham, B15 2TT UK; 2 Computational, Analytical and Structural Sciences, GlaxoSmithKline, Gunnels Wood Road, Stevenage, Hertfordshire, SG1 2NY UK; 3 Faculty of Medicine and Health Sciences, Stellenbosch University, Francie Van Zijl Dr, Parow, Cape Town, 7505 South Africa; 4 Division of Pharmacy and Optometry, School of Health Sciences, Manchester Academic Health Sciences Centre, University of Manchester, Manchester, M13 9PL UK; 5 Structural Genomics Consortium and Buchmann Institute for Molecular Life Sciences, Institute for Pharmaceutical Chemistry, Johann Wolfgang Goethe-University, Max-von-Laue-Straße 9, 60438 Frankfurt am Main, Germany; 6 Department of Biochemistry, University of Alberta, Edmonton, AB T6G 2H7 Canada

**Keywords:** Protein resonance assignment, NMR, Calcium/calmodulin dependent protein kinase, CaMK1D, CaMKIδ, CKLiK

## Abstract

The CaMK subfamily of Ser/Thr kinases are regulated by calmodulin interactions with their C-terminal regions. They are exemplified by Ca^2+^/calmodulin dependent protein kinase 1δ which is known as CaMK1D, CaMKIδ or CKLiK. CaMK1D mediates intracellular signalling downstream of Ca^2+^ influx and thereby exhibits amplifications of Ca^2+^signals and polymorphisms that have been implicated in breast cancer and diabetes. Here we report the backbone ^1^H, ^13^C, ^15^N assignments of the 38 kDa human CaMK1D protein in its free state, including both the canonical bi-lobed kinase fold as well as the autoinhibitory and calmodulin binding domains.

## Biological context

Protein kinases are important mediators of signal transduction, with approximately a 30% of all human proteins being phosphorylated (Cohen 2001). Their deregulation contributes to cancer and many other diseases, and is a major focus of drug discovery efforts (Marsden and Knapp 2008). To date, 518 members have been identified in the human kinome, and are grouped into 10 sub families (Manning et al. 2002). Those that belong to calmodulin dependent kinases (CaMK) group share a similar domain organization. All are activated by binding of Ca^2+^/calmodulin to their C-terminal regulatory region, releasing the catalytic domains to phosphorylate Ser/Thr residues in protein substrates to alter their functionality (Hook and Means 2001; Soderling and Stull 2001). No resonance assignments for any CaMK member have been reported, limiting analysis of their solution structures and interactions.

The structure of CaMK1D consists of various functional modules. The kinase domain resembles the canonical kinase fold first identified in PKA, which consists of a smaller β-sheet lobe which binds ATP connected by a hinge to the larger α-helical lobe where substrates are recognized. The β-sheet lobe features the P-loop and mobile αC helix, whilst the α-helical lobe presents the catalytic and activation loops (Knighton et al. 1991). Like other CaMK proteins, the kinase domain is negatively regulated by an adjacent C-terminal auto-inhibitory domain (AID), a helix-loop-helix motif which occludes the substrate and ATP binding sites (Goldberg et al. 1996; Swulius and Waxham 2008). The overlapping calmodulin binding domain (CBD) is recognized by calmodulin, which disengages the AID to activate the kinase (Haribabu et al. 1995; Yokokura et al. 1995; Matsushita and Nairn 1998).

Phosphorylation of Thr180 in the activation loop increases CaMK1D activity and is mediated by calcium calmodulin dependent protein kinase kinase (CaMKK). Diverse tissues express CaMK1D including brain, colon, liver, ovary, pancreas, prostate, spleen, testis, and thymus (Ishikawa et al. 2003). Its levels are amplified in basal-like breast tumors, which have a particularly poor clinical outcome. Engineered overexpression of CaMK1D in nontumorgenic cell lines causes increased cell proliferation, migration and invasion, indicating its oncogenic role (Bergamaschi et al. 2008). The subcellular distribution of CaMK1D is predominantly cytoplasmic, but once activated it can translocate to the nucleus (Sakagami et al. 2005). Increased protein expression levels and altered regulation of glucose processing have indicated a role for CaMK1D in diabetes (Fogarty et al. 2014; Haney et al. 2013).

The design of selective inhibitors and drug leads for kinases such as CaMK1D is challenging due to the high degree of conservation within the ATP binding pocket (Vulpetti and Bosotti 2004). NMR spectroscopy provides an efficient route for identifying efficient lead molecules, novel binding pockets and induced conformational changes (Diercks et al. 2001). However, protein kinases are challenging to study by NMR, often yielding poor spectra due to their intrinsic dynamics and large molecular weights. Thus few protein kinases have been assigned (Langer et al. 2004; Vogtherr et al. 2005, 2006; Gelev et al. 2006; Vajpai et al. 2008; Masterson et al. 2009; Xiao et al. 2015; Serimbetov et al. 2017; Sanfelice et al. 2018). Following optimization of solution conditions, the autoinhibited human CaMK1D was found to be amenable to NMR analysis, yielding resolved spectra under physiological buffer conditions. This information has been used to aid in the design of small molecule inhibitors of CaMK1D and development of lead candidates for therapeutic intervention (Fromont et al. 2020).

To enable investigation of CaMK1D’s solution structure and interactions, we have assigned the majority of the backbone resonances of CaMK1D using ^2^H, ^15^N and ^13^C-labelled protein.

## Methods and experimental

The construct of CaMK1D comprising the wild type human kinase catalytic domain and autoinhibitory domain (residues 1–333) included an N-terminal His_6_-tag. The *E. coli* BL21 DE3 RIPL codon plus strain (Stratagene) was transformed and grown at 37 °C in M9 minimal media supplemented with 30 μg/mL, kanamycin, 34 μg/mL chloramphenicol, ^15^NH_4_Cl and ^13^C_6_-glucose in 99.9% ^2^H_2_O until reaching an O.D 600_nm_ of 0.4. The culture was cooled to 18 °C and induced with 1 mM IPTG for 24 h. The cells were centrifuged at 7000 g for 15 min and resuspended in 50 mM Hepes pH 7.5, 500 mM NaCl, 0.5 mM TCEP, 5 mM imidazole, 0.02% NaN_3_ supplemented with an EDTA-free complete protease inhibitor cocktail (Roche). The cells were lysed by French press and the resulting cell lysate centrifuged at 75,000×*g* for 45 min. The supernatant was filtered through a 0.45 µM filter and CaMK1D was purified by Ni^2+^NTA-affinity chromatography (GE Healthcare). The His_6_-tag was removed with TEV protease followed again by purification on the Ni^2+^NTA-affinity column. Final purification was achieved by Superdex-75 (GE Healthcare) size exclusion chromatography in 50 mM Na phosphate pH 7.5, 150 mM NaCl, 0.5 mM TCEP, 0.02% NaN_3_, which indicated that the protein was monomeric. The CaMK1D sample was exchanged into an NMR buffer containing 50 mM Na phosphate pH 7.0, 75 mM NaCl, 0.5 mM TCEP, 0.02% NaN_3_ using an Amicon Ultra-15 centrifugal device (Millipore) and concentrated to 1.2 mM in a 600 µL final volume containing 10% ^2^H_2_O.

The NMR experiments were performed at 298 K on Varian Inova 800 and 900 MHz NMR spectrometers equipped with triple resonance cryogenic probes with Z-axis pulse field gradients. Backbone assignments were made from TROSY versions of ^1^H,^15^ N-HSQC, HNCO, HN(CA)CO, HN(CO)CA, HNCA, HNCACB and HN(COCA)CB (Gardner and Kay 1998). Spectra were processed with NMRPipe (Delaglio et al. 1995) and analysed using CCPN analysis (Vranken et al. 2005).

## Extent of assignments and data deposition

**Fig. 1:**
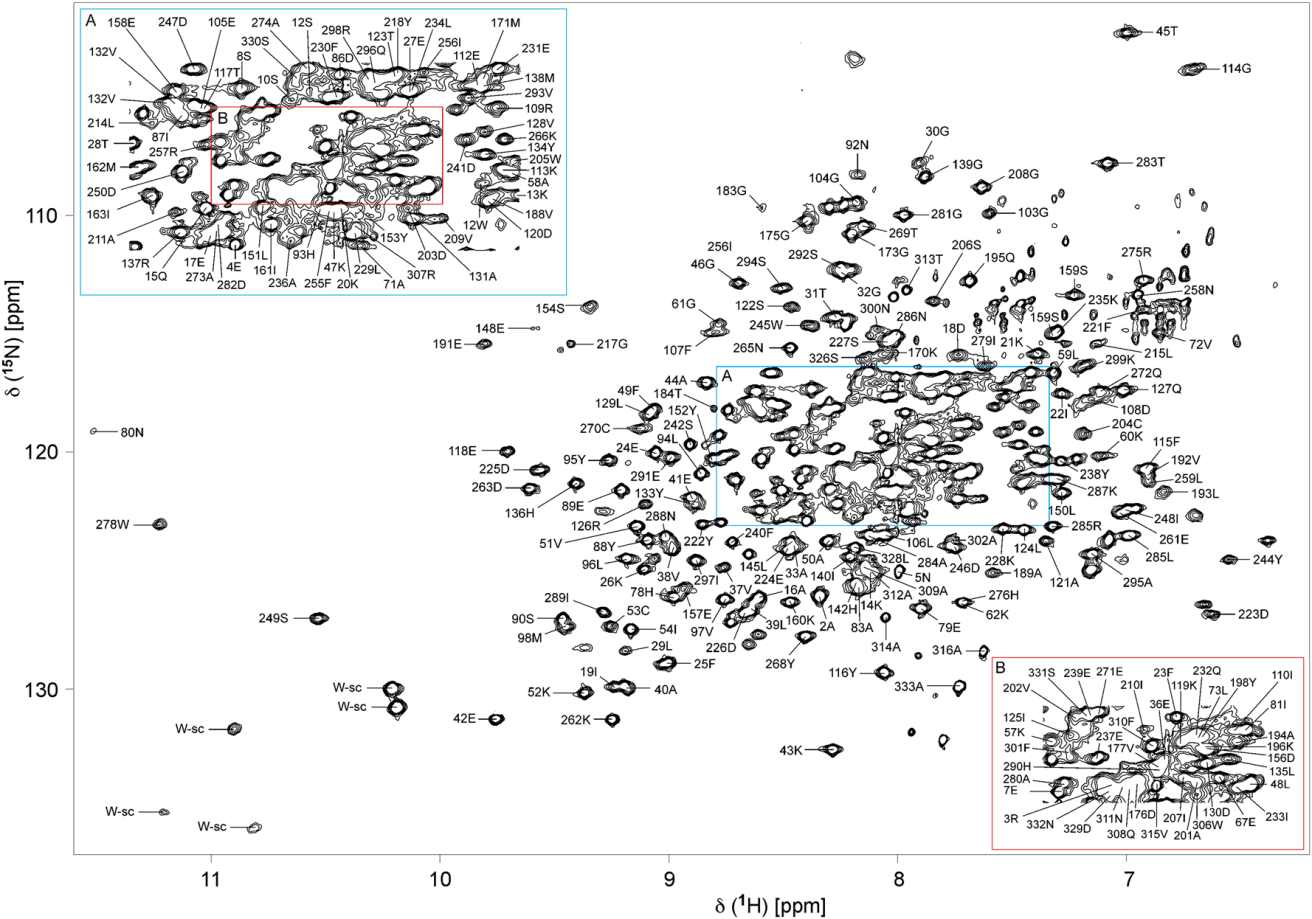
TROSY HSQC spectrum of deuterated CaMK1D at 1.2 mM concentration in 50 mM Na phosphate pH 7, 75 mM NaCl, 0.5 mM TCEP, 0.02% NaN_3_ collected at 298 K on a 800 MHz Varian Inova spectrometer. The blue box (denoted A) is an expansion of the crowded central region of the spectrum. The red box (denoted B) further expands the congested central region. Backbone HN peaks are labeled with their assignments. Trp sidechain HN groups are indicated by W-sc. Green boxes highlight examples of residues exhibiting more than one conformation

**Fig. 2:**
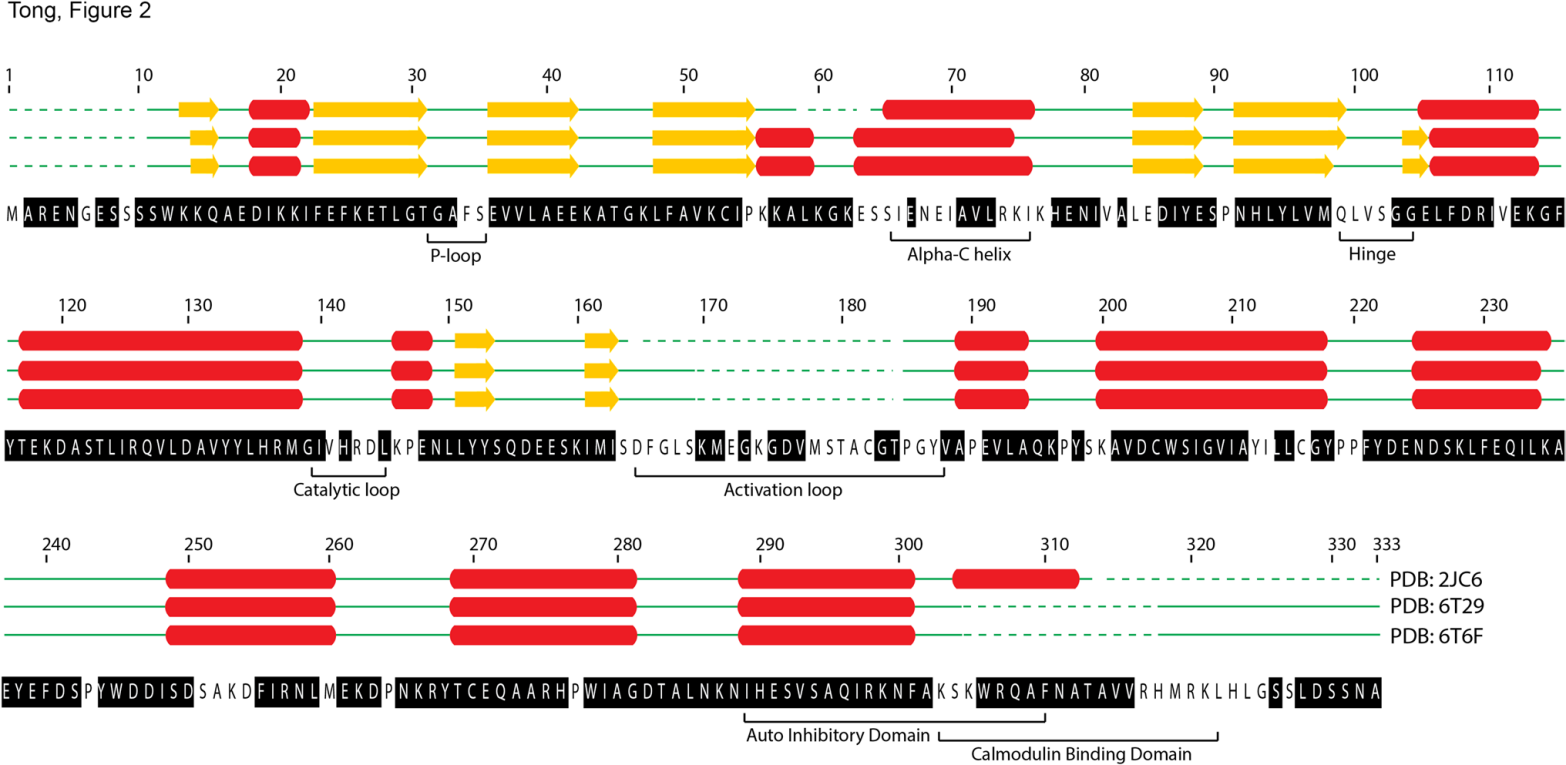
Backbone assignment coverage of the CaMK1D construct and the secondary structure deduced from the CaMK1D structure (PDBs 2JC6, 6T29, 6T6F). Assigned residues are highlighted in black in the sequence. Dotted green lines represent regions of the construct for which electron density could not be observed in the crystal structure. Solid green lines represent loops, yellow arrows are β-strands and red cylinders are α-helices

The ^1^H,^15^N-TROSY HSQC of the 37.8 kDa deuterated CaMK1D protein in its inactive state is shown (Fig. 1). The backbone HN resonances of 255 residues were assigned out of a possible 322, representing 79% coverage, and including residues from every structural and functional element (Fig. 2). Of those amino acids assigned, 98% include C’, 100% include Cα, and 99% include Cβ chemical shifts. Of all 11 proline residues, 10 were assigned, with 80% including C’, 60% including Cα and 40% including Cβ chemical shifts. The remaining Pro219 could not be assigned as it is N-terminal to another proline. Residues which could not be assigned were 1, 6, 9, 34–35, 56, 63–66, 68–70, 74–77, 82, 84–85, 99–102, 141, 143–144, 146, 164–169, 172, 174, 178–182, 186–187, 199–200, 212–213, 216, 251–254, 260, 303–305, 317–325 and 327. The unassigned residues are mostly found in loop regions, αC helix and C-terminus (Fig. 2). Incomplete assignments in the activation loop and C-terminus also coincided with the disorder and high B-factor values seen for these respective regions in the PDB (2jc6). Resonance assignments in these typically exposed and mobile elements were presumably compromised by peak broadening caused by intermediate or slow time scale dynamics. Assignments were also complicated by peak overlap near the center of the TROSY HSQC spectrum (Fig. 1). Several residues exhibited a second set of weaker or equivalent resonances, including for example Gly103, Gly217 and Asp223 suggesting additional conformers (Fig. 1, green boxes). The Ser residue at the extreme N-terminus which is retained from the TEV site after protease cleavage also could not be assigned. Isolated residue assignments were made for Glu67, Ala83, His142, Leu145 and Tyr 198 based on their unique chemical shifts, intra- and inter-residue correlations in the triple resonance spectra, and the CaMK1D structure.

The chemical shift values for the ^1^H, ^13^C and ^15^ N resonances of CaMK1D have been deposited at the BioMagResBank (https://www.bmrb.wisc.edu) under accession number.
